# Purification and Characterization of a Novel α-L-Rhamnosidase from *Papiliotrema laurentii* ZJU-L07 and Its Application in Production of Icariin from Epimedin C

**DOI:** 10.3390/jof8060644

**Published:** 2022-06-20

**Authors:** Hanghang Lou, Xiayu Liu, Siyu Liu, Qihe Chen

**Affiliations:** Department of Food Science and Nutrition, Zhejiang University, Hangzhou 310058, China; louhanghang@zju.edu.cn (H.L.); xiayuliu@zju.edu.cn (X.L.); siyuliu@zju.edu.cn (S.L.)

**Keywords:** *Papiliotrema laurentii* ZJU-L07, epimedin C, icariin, α-L-rhamnosidase, enzyme characteristics, optimization

## Abstract

Icariin is the most effective bioactive compound in Herba Epimedii. To enhance the content of icariin in the epimedium water extract, a novel strain, *Papiliotrema laurentii* ZJU-L07, producing an intracellular α-L-rhamnosidase was isolated from the soil and mutagenized. The specific activity of α-L-rhamnosidase was 29.89 U·mg^−1^ through purification, and the molecular mass of the enzyme was 100 kDa, as assayed by SDS-PAGE. The characterization of the purified enzyme was determined. The optimal temperature and pH were 55 °C and 7.0, respectively. The enzyme was stable in the pH range 5.5–9.0 for 2 h over 80% and the temperature range 30–40 °C for 2 h more than 70%. The enzyme activity was inhibited by Ca^2+^, Fe^2+^, Cu^2+^, and Mg^2+^, especially Fe^2+^. The kinetic parameters of *K_m_* and *V_max_* were 1.38 mM and 24.64 μmol·mg^−1^·min^−1^ using pNPR as the substrate, respectively. When epimedin C was used as a nature substrate to determine the kinetic parameters of α-L-rhamnosidase, the values of *K_m_* and *V_max_* were 3.28 mM and 0.01 μmol·mg^−1^·min^−1^, respectively. The conditions of enzymatic hydrolysis were optimized through single factor experiments and response surface methodology. The icariin yield increased from 61% to over 83% after optimization. The enzymatic hydrolysis method could be used for the industrialized production of icariin. At the same time, this enzyme could also cleave the α-1,2 glycosidic linkage between glucoside and rhamnoside in naringin and neohesperidin, which could be applicable in other biotechnological processes.

## 1. Introduction

As a kind of famous traditional Chinese herbal medicine, Herba Epimedii has been used for a long time in China because it could treat impotence and infertility, strengthen bone, and dispel rheumatism. The major active components of epimedium are prenyl-flavonoids including icariin, epimedin A, epimedin B, epimedin C, 2″-O-rhamnosyl icariside II, and baohuoside I [1]. The pharmacological effects of these flavonoids are different due to their different glycoside structures. Among them, icariin exhibits the most remarkable pharmacological activity. A number of studies have shown that the pharmacological effects of icariin include anti-inflammation [2], antiosteoporosis [3], antioxidative [4], antidepression [5], antitumors [6], neuroprotective effects [7], treating cardiovascular disease [8], and improving the reproductive system [9]. However, the content of icariin is rather limited in epimedium, which leads to the high price of icariin, and limits the application of icariin in medicine and functional foods.

To increase the production yield of icariin, the biosynthesis and bioconversion of icariin are gradually being studied. Biosynthesis of icariin from glucose or phenylpropionic acid has not been realized because it needs a series of reactions and some of these enzymes are not well-characterized. So far, it has been only reported that the biosynthesis of icaritin, the precursor of icariin, from glucose has been realized, and the biosynthesis of icariin needs further research [10]. Biotransformation of icariin gradually became the main method of preparing icariin, due to its fewer by-products, warm reaction condition, environment-friendly nature, and ease of completion. In the total flavonoids of epimedium, the structure of icariin is the most similar with epimedin C, which has a rhamnoside-α-1,2-rhamnoside at the C-3 position and has one more rhamnoside than icariin [11]. Generally, the content of epimedin C is higher than the other flavonol glycosides in some epimedium species such as *E. brevicornu*, and its content (15.96 mg·g^−1^) could even be 3–5 times that of icariin (5.36 mg·g^−1^) in the root of *E. brevicornu* [12]. Therefore, epimedin C could be the best substrate for the biotransformation of icariin, while α-L-rhamnosidase is an important tool in the process of biotransformation. At present, some studies have shown that epimedin C could be converted into icariin by enzymatic hydrolysis of α-L-rhamnosidase. α-L-rhamnosidase from *Bacteroides thetaiotaomicron* has the ability to transform epimedin C into icariin, and the bioconversion of epimedin C into icariin could reach 90.5% under the optimum conditions [13]. Identically, it was reported that recombinant *Escherichia coli* cells expressing α-L-rhamnosidase from *Aspergillus nidulans* could hydrolyze epimedium extracts to prepare icariin [14].

α-L-Rhamnosidase (3.2.1.40) can release the terminal L-rhamnose from a series of glycosides, glycolipids, and some natural products by cleaving α-1,2, α-1,3, α-1,4, or α-1,6 glycosidic bonds [15]. This enzyme has been found in animal tissue, plants, yeasts, bacteria, and fungi, among them bacteria and fungi are the main producing sources. α-L-Rhamnosidase is an important biotechnology enzyme and has many applications in the pharmaceutical and food industries. For example, α-L-rhamnosidase from *Alternaria alternata* SK37.001 can make citrus juices less bitter [16], and α-L-rhamnosidase from *Aspergillus niger* can improve wine aromas [17]. Moreover, α-L-rhamnosidase can also manufacture L-rhamnose [18] and prepare the key precursor of food additives such as quercetin-3-β-D-glucopyranoside and hesperetin 7-O-glucoside [19,20].

In recent decades, α-L-rhamnosidase-producing fungi and bacteria have been widely studied. However, the studies about the enzyme from yeasts are few. In this study, a strain producing an intracellular α-L-rhamnosidase was screened and identified as *Papiliotrema laurentii* ZJU-L07. Meanwhile, we aimed to purify and characterize a new intracellular α-L-rhamnosidase and to research whether α-L-rhamnosidase has potential applications in hydrolyzing some natural products. The enzyme showed great catalytic activity specifically towards the rhamnoside-α-1,2-rhamnoside glycosidic linkage at the C-3 position in epimedin C. To improve the efficiency of the bioconversion of epimedin C into icariin and increase the content of icariin in the epimedium water extract, we also conducted the optimization of the enzymatic hydrolysis condition.

## 2. Materials and Methods

### 2.1. Materials and Chemicals

Soil samples used for the strain screening were collected from Herba Epimedii botanical garden in Huangshi, China. Epimedin C, prunin, and hesperetin-7-O-glucoside were obtained from Chengdu Desite Biotechnology Co., Ltd. (Chengdu, China). Icariin, neohesperidin, hesperidin, naringin, and isoquercitrin were purchased from Aladdin (Shanghai, China). Rutin was purchased from Shanghai Macklin Biochemical Technology Co., Ltd. (Shanghai, China). *p*-nitrophenyl-α-L-rhamnopyranoside (pNPR) was bought from Nantong Feiyu Biological Technology Co., Ltd. (Nanjing, China). Modified BCA Protein Assay Kit and protein markers for SDS-PAGE were produced from Sangon Biotech Engineering Co., Ltd. (Shanghai, China). HPLC-grade acetonitrile was produced from Tianjin Siyou Fine Chemical Co., Ltd. (Tianjin, China). Epimedium water extract (EWE) was provided from Jing Brand Chizhengtang Pharmaceutical Co., Ltd. (Huangshi, China). Two kinds of epimedium water extract were used in this work, one kind of epimedium water extract contained 50% of epimedin C, and another kind of epimedium water extract contained 20% of epimedin C. To make it easier to distinguish, the former was named EWE-I and the latter was named EWE-II. Other chemicals were of reagent grade.

### 2.2. Strain Screening, Mutagenesis, and Identification

The strain, producing α-L-rhamnosidase specifically for bioconverting epimedin C into icariin, was isolated from soil samples growing *E. brevicornu*. The soil sample was dissolved in 25 mL of the sterilized liquid medium having the composition: (g·L^−1^): EWE-II 20. The media were cultured at 30 °C, 180 rpm. After 24 h of incubation, the fermentation solution was diluted and then seeded into the solid medium of the first screening (g·L^−^^1^): EWE-II 20, agar 20. Then, the strains were picked up and plated out on the liquid medium of the secondary screening, which contained (g·L^−1^): epimedin C 0.05, sucrose 30, NaNO_3_ 3, KCl 0.5, K_2_HPO_4_·3H_2_O 1, MgSO_4_·7H_2_O 0.5, and FeSO_4_ 0.01 (natural pH). The target strain was determined by whether icariin was produced. The content of icariin was analyzed using a HPLC system. In order to increase enzyme activity, various intensities of gamma rays (60 Co^γ^, 250 Gy, 500 Gy, 750 Gy, 1000 Gy) and different concentrations of nitrosoguanidine (0.5, 1, 1.5, 2 M) were applied to mutagenesis of the target strain [21]. After mutagenesis, Luria Bertani (LB) medium including pNPR was used on the first screening. The selected strains were inoculated into the medium of the secondary screening, which contained (g·L^−1^): epimedin C 0.05, NaNO_3_ 6, peptone 6, yeast extract 6, CaCl_2_ 0.1, K_2_HPO_4_ 0.1, and FeSO_4_·7H_2_O 0.1. The strain with the highest yield of icariin was isolated.

Then, the total DNA of the strain was extracted using Puregene^®^ DNA Handbook. The ITS sequence was amplified by PCR using primers ITS1(5′-TCCGTAGGTGAACCTGCGG-3′) and ITS4(5′-TCCTCCGCTTATTGATATGC-3′). The genotypic identification of the strain was completed by analyzing the ITS sequence using the BLAST program (NCBI, Bethesda, MD, USA). The physiological and biochemical identification of the strain was finished in the Zhejiang Institute of Microbiology using Merieux VITEK2 compact automatic microbial analysis system. The neighbor-joining tree was drawn using MEGA-X.

### 2.3. Preparation and Purification of α-L-Rhamnosidase

The strain was cultivated aerobically at 28 °C for 4 d in a medium containing 1% L-rhamnose, 1% NaNO_3_, 0.3% yeast extract, 0.3% peptone, 0.001% K_2_HPO_4_, 0.001% FeSO_4_·7H_2_O, and 0.001% CaCl_2_. After liquid cultivation, the cells were collected by centrifugation at 2328× *g* for 10 min, washed twice with distilled water, resuspended in phosphate buffer (0.1 M, pH 7.4), with ultrasonic crushing for 10 min at a low temperature (<10 °C). Then, the cellular extracts were centrifuged at 6790× *g*, 4 °C for 10 min, and the supernatants were considered as crude enzyme.

α-L-Rhamnosidase was obtained after purification of the crude enzyme. In order to maintain enzyme activity, all the purification steps were finished at 4 °C. First, the crude enzyme was mixed with ammonium sulfate salt up to 30% saturation, and the mixture was kept for 4 h and centrifuged to remove protein impurities. The ammonium sulfate was added to the supernatant to achieve a final concentration of 70% saturation. Second, the mixture was centrifuged at 10,611× *g*, 4 °C for 10 min, and the precipitate was resuspended and dialyzed in citric acid-Na_2_HPO_4_ buffer (pH 7.0). The enzyme solution was concentrated by using PEG 6000 and separated on a Sephacryl S-200 gel filtration column (Φ1.75 × 43 cm). Finally, the elution was completed with the citric acid-Na_2_HPO_4_ buffer (50 mM, pH 7.0) at a flow rate of 0.6 mL·min^−1^. The fractions with higher α-L-rhamnosidase activity were collected, concentrated and purified further using a DEAE Sepharose^TM^ Fast Flow column (Φ1.6 × 20 cm) from Shanghai Chenqiao Biotechnology Co., Ltd. (Shanghai, China). The column was eluted with a NaCl linear gradient elution (0–1.0 M) in citric acid-Na_2_HPO_4_ buffer (50 mM, pH 7.0) at a flow rate of 0.6 mL·min^−1^. Collected fractions were screened for assaying protein concentration and α-L-rhamnosidase activity. Active fractions were combined, dialyzed in citric acid-Na_2_HPO_4_ buffer (pH 7.0), and concentrated by using PEG 6000. The molecular weight of the purified enzyme was detected by Polyacrylamide gel electrophoresis (SDS-PAGE) [22].

### 2.4. Assay of α-L-Rhamnosidase Activity and Protein Concentration

The activity of α-L-rhamnosidase was assayed using the method described previously [16]. Amounts of 50 μL of 10 mM pNPR and 400 μL of citric acid-Na_2_HPO_4_ buffer (200 mM, pH 7.0) were mixed and preheated for 5 min in 55 °C. Then, 50 μL of the enzyme solution was added to the mixture and incubated at 55 °C for 10 min. The reaction was stopped by adding 750 μL of 1.5 M Na_2_CO_3_. The liberated *p*-nitrophenyl was determined at 405 nm by a microplate reader. One unit (U) of α-L-rhamnosidase was defined as the amount of enzyme liberating 1 μmol of *p*-nitrophenol per min at 55 °C and pH 7.0. The protein concentration was quantified using a modified BCA Protein Assay Kit with bovine serum albumin as the standard.

### 2.5. Determination of Enzymatic Characteristics

#### 2.5.1. Optimal Temperature and Thermal Stability

pNPR as the substrate was used to determine the optimum temperature of α-L-rhamnosidase. The reaction mixture was incubated at different temperatures (30–70 °C) in the citric acid-Na_2_HPO_4_ buffer (200 mM, pH 7.0) for 10 min [13]. The highest enzyme activity was set as 100%. To evaluate the temperature stability, the enzyme was kept in the citrate-phosphate buffer (200 mM, pH 7.0) at different temperatures (30, 35, 40, 45, 50, 55, 60, 65, and 70 °C) for various times (20, 40, 60, 80, and 120 min) [14]. Then, the enzyme activity was assayed as described in Section 2.4. The activity of the enzyme without preincubation was set as 100%.

#### 2.5.2. Optimal pH and pH Stability

The optimum pH of α-L-rhamnosidase was measured by incubating the reaction mixture at 55 °C in the pH range of 4.0–9.0 for 10 min, using pNPR as the substrate [13]. The highest enzyme activity was set as 100%. To evaluate the pH stability, the α-L-rhamnosidase was incubated in the buffers from pH 4.0 to 9.0 at 28 °C for 2 h. Then, the method as described in Section 2.4 was used to assay enzyme activity. The buffers used were 200 mM citrate-phosphate buffer (pH 4.0–7.0) and 50 mM Tris-HCl (pH 7.5–9.0) [13]. The activity of the enzyme without preincubation was set as 100%.

#### 2.5.3. The Effects of Metal Irons on the α-L-Rhamnosidase Activity

To study the effect of metal ions on α-L-rhamnosidase activity, Ca^2+^, Cu^2+^, Fe^2+^, Mg^2+^, Mn^2+^, Ni^2+^, Co^2+^, Ba^2+^, K^+^ and Al^3+^ were added to the reaction system to make the final concentration of 0.2 mM [16]. The enzyme activity was measured as described above and the relative activity without adding metal ions was set as 100%.

#### 2.5.4. Kinetic Parameters

The kinetic parameters of α-L-rhamnosidase were investigated by using different concentrations of pNPR (2.0–10.0 mM) as artificial substrate and different concentrations of epimedin C (1.0–5.0 mM) as natural substrate [16]. The maximum rate (*V_max_*) and Michaelis constant (*K_m_*) were computed from Lineweaver–Burk plots.

### 2.6. Substrate Specificity

The substrate specificity of the enzyme was analyzed by using naringin, neohesperidin, epimedin C, hesperidin, and rutin as substrates. A total of 1 g·L^−1^ of 200 μL substrates and 100 μL enzyme solution were mixed and reacted at the optimum conditions for 6 h. The reaction was stopped by ice bath and adding 900 μL methanol. Then, the mixtures werecentrifuged at 6790× g for 5 min. The supernatants were filtered using a 0.22 μm nylon membrane ahead of detecting released products via high performance liquid chromatography (HPLC).

### 2.7. Enzymatic Hydrolysis Optimization of Epimedin C

Standard epimedin C, EWE-I, and EWE-II were each hydrolyzed by the crude enzyme solution at 50 °C. The concentration of epimedin C in the reaction systems was 500 mg·L^−1^. The concentration of epimedin C and icariin was determined by HPLC with incubation for 0.5, 1, 2, 4, 6, and 8 h. The effects of different purities of epimedin C on bioconversion of epimedin C to icariin were investigated according to icariin yield and epimedin C remaining. The calculation method of icariin yield and epimedin C remaining is in the Supplementary Materials.

To increase the content of icariin in the EWE-II, enzymatic hydrolysis conditions were optimized. Firstly, the concentration of epimedin C (500 mg·L^−1^, 1000 mg·L^−1^, 1500 mg·L^−1^), the amount of α-L-rhamnosidase (2.10 U, 3.95 U, 8.79 U, 19.13 U in 2 mL of the reaction volume), reaction temperature (40 °C, 45 °C, 50 °C, 55 °C) and pH (6, 6.5, 7) were selected for single factor experiments. Secondly, the yield of icariin and epimedin C remaining were used as indexes to investigate the influence of the four single factors on the preparation of icariin by biological enzymatic method. Then, the important factors and levels on icariin yield were determined and the production of icariin was optimized by response surface methodology (RSM). The Design Expert software for Box–Behnken Design was used to design a response surface analysis test with three factors and three levels, with the yield of icariin and epimedin C remaining as the response values. The experimental design is shown in Table 1, where *X*_1_, *X*_2_, and *X*_3_ are the values of enzyme dosage, pH, and time, respectively, and *Y*_1_ and *Y*_2_ represent the values of the yield of icariin and epimedin C remaining, respectively. In the single factor experiments and response surface methodology, 1 mL of EWE-II and 1 mL of the crude enzyme were mixed to react, and the reaction volume was always 2 mL.

### 2.8. HPLC Analysis

The flavonoids were determined by HPLC analysis using the modified methods described previously [13,16,20,23]. The epimedin C and icariin were analyzed using an HPLC system (Angilent1100) and a C18 column with acetonitrile (solvent A) and water (solvent B). The sample volume injected was 10 μL and the column temperature was 30 °C. The flow rate was 1 mL·min^−1^ and the absorbance was determined at 270 nm [13,23]. For the elution program, the following proportions of solvent A were used: 0–15 min, 22%A–28%A; 15–30 min, 28%A–29%A; 30–56 min, 29%A–100%A. Other substrates and hydrolysis products were detected using an HPLC system (Agilent1100) and a C18 column with acetonitrile (solvent A) and water (solvent B). The sample volume injected was 10 μL and the flow rate was 1 mL·min^−1^. The column temperature was 40 °C and the absorbance was determined at 280 nm [16,20]. For the elution program, the following proportions of solvent A were used: 0–3 min, 25%A; 3–18 min, 25%A–32%A; 18–22 min, 32%A–100%A; 22–26 min, 100%A.

### 2.9. Statistical Analysis

In the optimization process, at least three independent experiments were conducted for the purpose of standard error analysis of the mean. The Design-Expert 6.0 trial version was applied to design optimized experiments and perform regression analysis on the experimental data [24].

## 3. Results

### 3.1. Strain Screening and Identification

**Figure 1:**
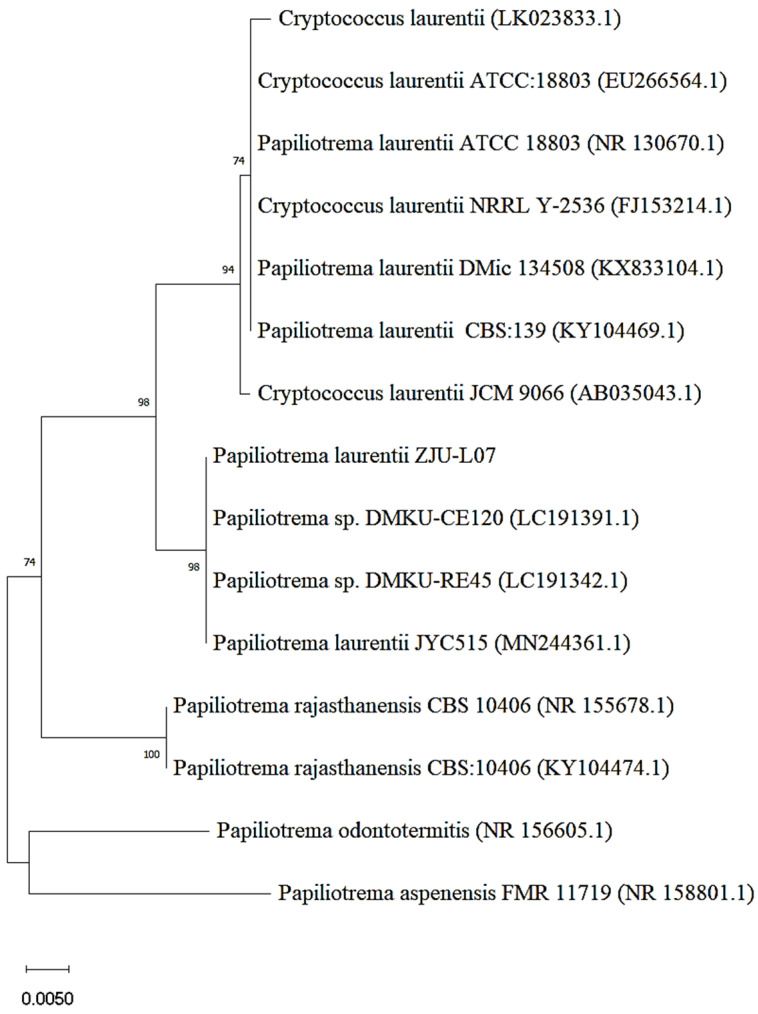
Neighbor-joining tree of *P. laurentii* ZJU-L07.

The strain with bioconversion of epimedin C into icariin was obtained through two screening tests from the soil samples. After mutagenesis with gamma rays and nitrosoguanidine, 16 strains were screened out according to the degree of yellowing of the medium including pNPR (Figure S1a). Then, the strain with the highest yield of icariin was picked up from the 16 strains (Figure S1b). The morphology of this strain on yeast extract peptone dextrose (YPD) medium is presented in Figure S1c. Using ITS analysis and a BLAST search, this strain was identified as *Papiliotrema laurentii*. The isolated strain was identified as *Cryptococcus* *laurentii* by the physiological and biochemical identification. It was reported that taxonomic revision of the basidiomycete genus *Cryptococcus* caused *C. laurentii* to be renamed as *P. laurentii* [25]. Therefore, the strain was identified as *Papiliotrema laurentii* and designated as *P. laurentii* ZJU-L07 in this work. The phylogenetic relationship between *P. laurentii* ZJU-L07 and other *Papiliotrema* (*Cryptococcus*) species is shown by a neighbor-joining tree (Figure 1). The strains which *P. laurentii* -L07 has close phylogenetic relationship with are *Papiliotrema* sp. DMKU-CE120, *Papiliotrema* sp. DMKU-RE45, and *P*. *laurentii* JYC515.

### 3.2. Purification of α-L-Rhamnosidase from P. laurentii ZJU-L07

**Figure 2:**
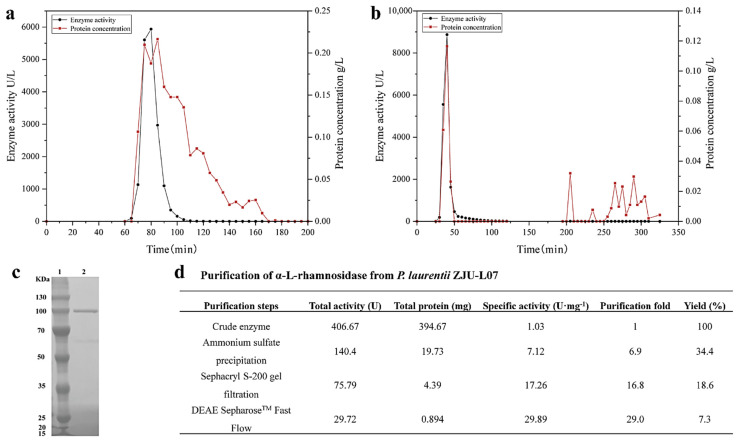
(**a**) Sephacryl S-200 gel chromatography of α-L-rhamnosidase. (**b**) DEAE SepharoseTM Fast Flow gel chromatography of α-L-rhamnosidase. (**c**) SDS-PAGE analysis of the purified enzyme. Lane 1, the protein marker; lane 2, the purified enzyme. (**d**) Purification of α-L-rhamnosidase from *P. laurentii* ZJU-L07.

The intracellular α-L-rhamnosidase from *P. laurentii* ZJU-L07 was purified step-by-step. A summary of the purification steps was presented in Figure 2d. After incubation, the cells were collected, broken by sonication, and centrifugated, the crude enzyme was obtained, and its specific activity was 1.03 U·mg^−1^ of protein. After ammonium sulfate precipitation, the specific activity was 7.12 U·mg^−1^ of protein, increasing 6.9-fold. Then, the specific activity was raised to 17.26 and 29.89 U·mg^−1^ of protein, respectively, by gel filtration chromatography (Figure 2a) and anion exchange chromatography (Figure 2b). The final purification resulted in a 29-fold increase in specific activity and a level of recovery of 7.3% for α-L-rhamnosidase. The specific activity of α-L-rhamnosidase from *P. laurentii* ZJU-L07 was higher than that of α-L-rhamnosidase from *Alternaria alternata* SK37.001 (21.7 U·mg^−1^ of protein) [16] and similar to that of α-L-rhamnosidase from *Pichia angusta* X349 (33.9 U·mg^−1^ of protein) [26]. The purity of the purified enzyme was examined by SDS-PAGE analysis. The result is shown in Figure 2c. The molecular weight markers were located in lane 1 and the purified enzyme was located in lane 2. SDS-PAGE analysis indicated the molecular mass of α-L-rhamnosidase was 100 kDa. Molecular masses of α-L-rhamnosidases have been reported to range from 53.0 to 240 kDa [15]. The molecular mass of the enzyme from *P. laurentii* ZJU-L07 was similar to those of α-L-rhamnosidases from *Bacillus* sp. GL1 (100.0 kDa) [27], *Bifidobacterium dentium* (100.0 kDa) [28], *Thermotoga petrophila* (101.7 kDa) [23], *Aspergillus nidulans* (102.0 kDa) [29], and *Penicillium griseoroseum* MTCC-9224 (97.0 kDa) [30].

### 3.3. Enzymatic Characterization

#### 3.3.1. Optimum Temperature and Thermal Stability

**Figure 3:**
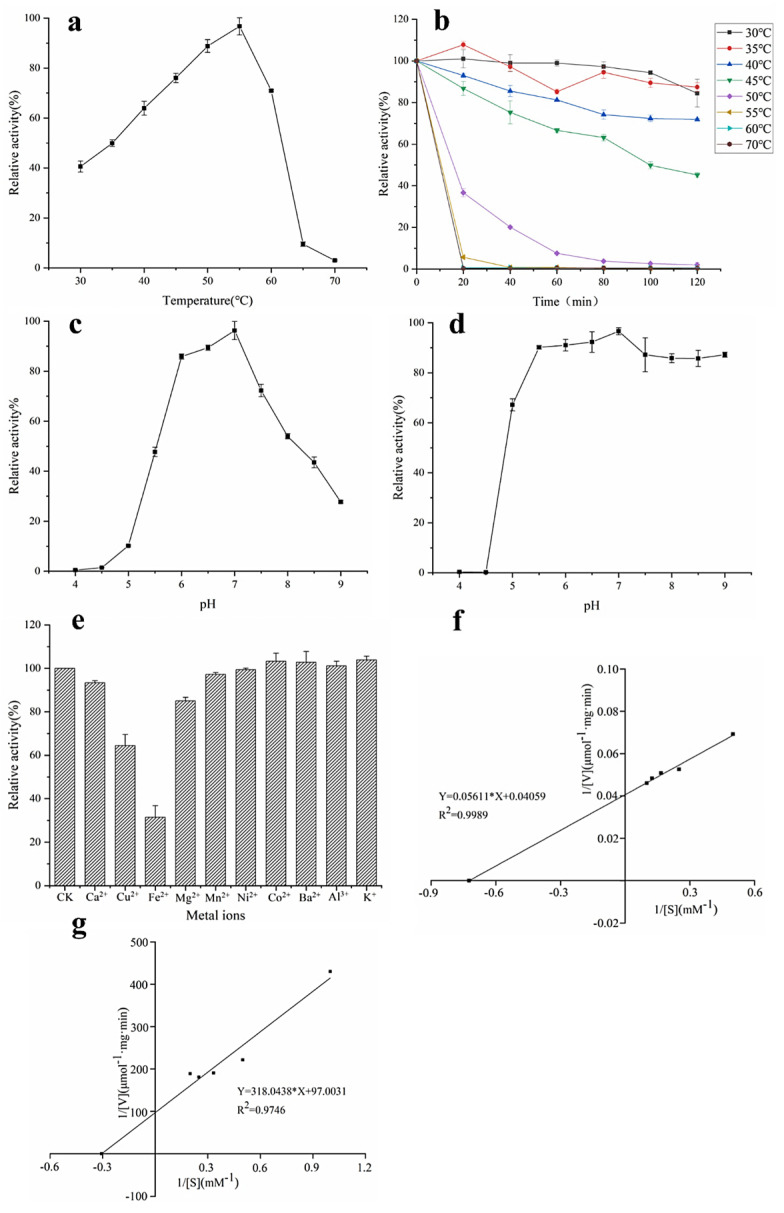
Properties of α-L-rhamnosidase. (**a**) Optimum temperature, (**b**) thermal stability, (**c**) optimum pH, (**d**) pH stability, (**e**) effects of metal ions, (**f**) kinetic parameters with pNPR as substrate, and (**g**) kinetic parameters with epimedin C as substrate.

The catalyzed reaction rate of the enzyme is closely related to temperature, so temperature is an important factor in the study of the enzymatic properties of α-L-rhamnosidase. The optimal temperature of α-L-rhamnosidase was determined to be 55 °C (Figure 3a). Figure 3b demonstrated that α-L-rhamnosidase was stable from 30 °C to 40 °C, and the residual enzyme activity was still more than 70% after incubation for 120 min. When the enzyme was kept in 45 °C for 120 min, the residual enzyme activity was about 50%. Its residual activity was less than 10% after being incubated above 55 °C for 20 min. Therefore, the enzyme was sensitive to temperature, and unsuitable for enzymatic hydrolysis under high temperature for a long time.

#### 3.3.2. Optimal pH and pH Stability

pH is also another important factor affecting enzyme activity and the catalyzed reaction rate of the enzyme. It could be seen from Figure 3c that the optimal pH of α-L-rhamnosidase was 7.0 and its activity was higher than 70% of the maximum activity in the pH range 6.0–7.5. pH stability assays indicated that α-L-rhamnosidase was stable at a pH range of 5.5–9.0 with an activity of over 80% (Figure 3d).

#### 3.3.3. Effects of Metal Ions on Enzyme Activity

The effects of metal ions on α-L-rhamnosidase activity were shown in Figure 3e. Most of the metal ions had no obvious activation effect on α-L-rhamnosidase activity in the final concentration of 0.2 mM, while the Ca^2+^, Fe^2+^, Cu^2+^, and Mg^2+^ showed inhibition on α-L-rhamnosidase. The relative activity decreased to 31.4% and 64.4% with the addition of Fe^2+^ and Cu^2+^, respectively. Ca^2+^ and Mg^2+^ had a slight inhibitory effect, reducing 6.4% and 15% of α-L-rhamnosidase activity, respectively.

#### 3.3.4. Kinetic Parameters

When pNPR was chosen as an artificial substrate to determine the kinetic parameters of α-L-rhamnosidase, the values of *K_m_* and *V_max_* were 1.38 mM and 24.64 μmol·mg^−1^·min^−^^1^, respectively (Figure 3f), as calculated from the Lineweaver−Burk plots. *K_m_* means the affinity of enzyme and substrate, and the larger the value of *K_m_* means the smaller the affinity between enzyme and substrate. *K_m_* of α-L-rhamnosidase studied with pNPR as the substrate was lower than those of α-L-rhamnosidase from some other microorganisms, which indicated the affinity of the enzyme for pNPR was superior to that of other-sourced enzymes [13,16,31]. When epimedin C was used as a nature substrate to determine the kinetic parameters of α-L-rhamnosidase, the values of *K_m_* and *V_max_* were 3.28 mM and 0.01 μmol·mg^−1^·min^−1^, respectively (Figure 3g). *K_m_* of α-L-rhamnosidase with epimedin C as the substrate was higher than those of α-L-rhamnosidase from *A. nidulans* and *T. petrophila* [14,23].

### 3.4. Substrate Specificity

α-L-Rhamnosidase is a hydrolase that can specifically cleave terminal rhamnose from a large number of natural products. The substrate specificity of the enzyme from *P. laurentii* ZJU-L07 was evaluated by hydrolyzing epimedin C, neohesperidin, naringin, hesperidin, and rutin. The enzyme could convert epimedin C, neohesperidin, naringin, hesperidin, and rutin into icariin, hesperetin-7-O-glucoside, prunin, hesperetin-7-O-glucoside, and quercetin-3-β-D-glucoside, respectively, but the yields of these products were different. As could be seen from Table 2, the hydrolysis effect of the enzyme on epimedin C, neohesperidin, and naringin was superior to hesperidin and rutin. These results illustrated that the enzyme had higher selectivity to cleave the α-1,2 glycosidic linkage between glucoside and rhamnoside and the α-1,2 glycosidic linkage between rhamnoside and rhamnoside than to cleave the α-1,6 glycosidic linkage between glucoside and rhamnoside. The high-performance liquid chromatography of substrates and products is shown in Figure S2.

### 3.5. Enzymatic Hydrolysis Optimization of Epimedin C by α-L-Rhamnosidase

#### 3.5.1. Selection of the Suitable Substrate

**Figure 4:**
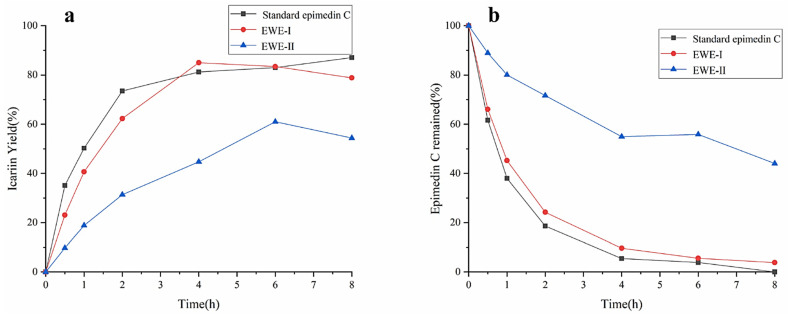
The effects of different purities of epimedin C on the yield of icariin (**a**) and epimedin C remaining (**b**).

The effects of different purities of epimedin C on the yield of icariin were investigated using standard epimedin C, EWE-I, and EWE-II as the substrates. The result was shown in Figure 4a,b. No matter what kind of substrate was used, the yield of icariin increased gradually, and the amount of epimedin C gradually decreased with the increase in reaction time. The maximum yield of icariin reached 87% at 8 h with standard epimedin C as substrate, while epimedin C was almost entirely utilized. The result manifested that icariin was derived from the transformation of epimedin C, and the other two substrates also had the same situation during the hydrolysis process (icariin yield% < 100%-epimedin C remaining%). When reacting for 4 h with EWE-I as the substrate, the yield of icariin reached 85%. However, as EWE-II and crude enzyme were mixed with incubating for 6 h, the yield of icariin was only 61%, which was about 25% lower than the other two substrates. At 8 h, about 40% of epimedin C was not hydrolyzed. It could be seen from the above results that when the concentration of epimedin C in the reaction system was identical, the higher the purity of epimedin C in the substrate, the higher the yield of icariin. However, *E*. *wanshanense* Ying water extract (EWE) containing lower levels of epimedin C was easy to obtain in practical production because of its cheapness and simple production process. Therefore, in order to improve the content of icariin, EWE-II was selected as the substrate to optimize the enzymatic conditions.

#### 3.5.2. Screening of Enzymatic Conditions Using the Single Factor Test

**Figure 5:**
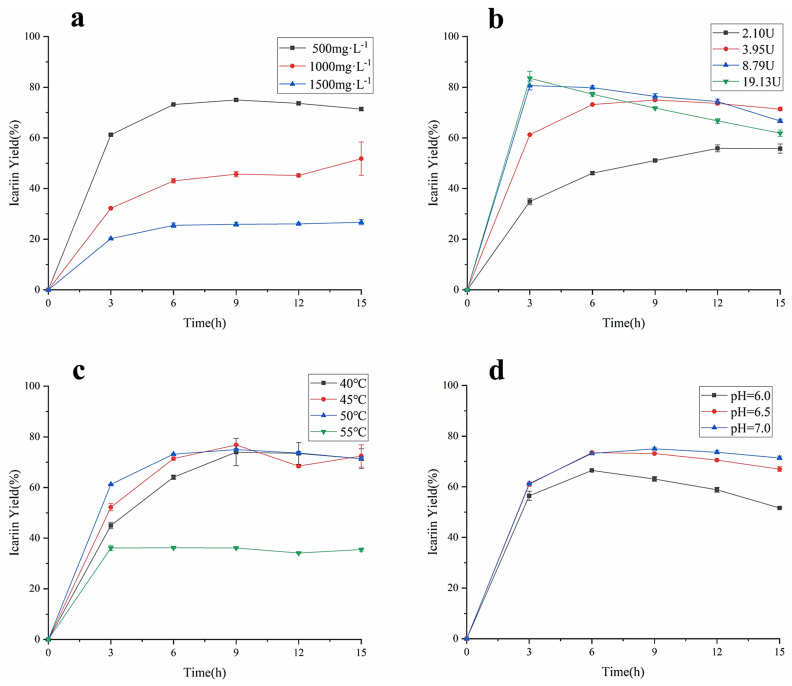
(**a**) The amount of α-L-rhamnosidase on icariin yield at 50 °C under pH 4.0 with 3.95 U of enzyme. (**b**) The amount of α-L-rhamnosidase on icariin yield at 50 °C under pH 4.0 with 500 mg·L^−1^ epimedin C. (**c**) Temperature influence with 3.95 U of enzyme in the reaction system containing 500 mg·L^−1^ epimedin C at pH 7.0. (**d**) pH influence with 3.95 U of enzyme in the reaction system containing 500 mg·L^−1^ epimedin C at 50 °C.

As the important factors affecting the enzyme-catalyzed reaction rate, the concentration of epimedin C, the amount of α-L-rhamnosidase, the reaction temperature, and the pH were studied for their effect on the yield of icariin and epimedin C remained. As shown in Figure 5a, when the other reaction conditions were the same, the yield of icariin decreased as the concentration of epimedin C increased, which was similar to the previous study [14]. The biggest icariin yield in the reaction system containing 500 mg·L^−1^ epimedin C was 74.99 ± 0.42%, the value was 51.81 ± 6.56% and 26.67 ± 1.02% in the reaction system loaded 1000 mg·L^−1^ epimedin C and 1500 mg·L^−1^ epimedin C, respectively. However, regardless of the concentration of epimedin C in the reaction system, the conversion rate of epimedin C was the fastest before 3 h of incubation, and the yield of icariin did not show an upward trend after 9 h of incubation, which means α-L-rhamnosidase basically stopped working. To verify the effect of enzyme activity on the yield of icariin, different amounts of α-L-rhamnosidase were incubated with EWE-II (Figure 5b). When the enzyme activity reached 2.10 U, 3.95 U, 8.79 U, and 19.13 U, the highest conversion rate was 55.88 ± 1.02%, 74.99 ± 0.42%, 80.67 ± 1.73%, and 83.56 ± 2.73%, respectively. These results revealed that the higher the amount of enzyme in the reaction system, the higher the yield of icariin. However, with the increase in enzyme activity, the yield of icariin increased more slowly. When the reaction time was 3 h, the yield of icariin adding 8.79 U amount of enzyme was significantly different from that of adding 2.10 U amount of enzyme and adding 3.95 U amount of enzyme, but not much different from that at enzyme activity 19.13 U. It showed that when the amount of substrate was constant, the higher the amount of enzyme, the more the enzyme bonded to the substrate. Upon the binding of enzyme and substrate reaching saturation, even if the amount of enzyme was increased, the yield would no longer increase. After 3 h of incubation, the yield of icariin decreased when the enzyme amount was 8.79 U and 19.13 U. Figure 5c indicates the yield of icariin under four levels of temperature: 40, 45, 50, and 55 °C. The enzymatic hydrolysis of EWE-II was restrained under 55 °C with a much lower the yield of icariin than 40, 45, or 50 °C. Before 7 h of incubation, the bioconversion rate of epimedin C was proportional to temperature no more than 50 °C, while the yield of icariin at 45 °C was higher than the yield of icariin at 40 °C and 50 °C after 7 h of incubation. The biggest icariin yield was 76.79 ± 0.28% at 45 °C for 9 h. In general, the reaction temperature had a great impact on the yield of icariin and a mild temperature of 45 °C was applicable for the enzymatic hydrolysis of epimedin C. As can be seen from Figure 5d, the yield of icariin increased with the increase in pH. When the value of pH was 7, the maximum yield of icariin was 74.99 ± 0.42% for 9 h. Compared to an acidic environment, α-L-rhamnosidase was more suitable for enzymatic hydrolysis of epimedin C under a neutral environment. At the same time, the bioconversion of epimedin C in a weakly alkaline environment was worth investigating. As for the changes in the content of epimedin C, there was a similar trend compared with the content of icariin (Figure S3). In the single factor test, when EWE-II was hydrolyzed at 50 °C under pH 7.0 by 19.13 U of α-L-rhamnosidase and the concentration of epimedin C was 500 mg·L^−1^ in the reaction system, the yield of icariin reached the highest (83.56%) and the residual of epimedin C was 5.05%.

In view of the above results in Figure 5a–d, the activity of α-L-rhamnosidase and pH were selected for further optimization of enzymatic conditions. The yield of icariin decreased as the concentration of epimedin C rose, and the concentration of epimedin C was identified as 500 mg·L^−1^. The optimal temperature of epimedin C degradation was determined at 45 °C. As the four factors were optimized, it was found that the optimal yield appeared at different time points. The reaction time was also an important factor and set as a factor for further optimization.

#### 3.5.3. Optimization of Icariin Yield and Epimedin C Remaining with RSM

**Figure 6:**
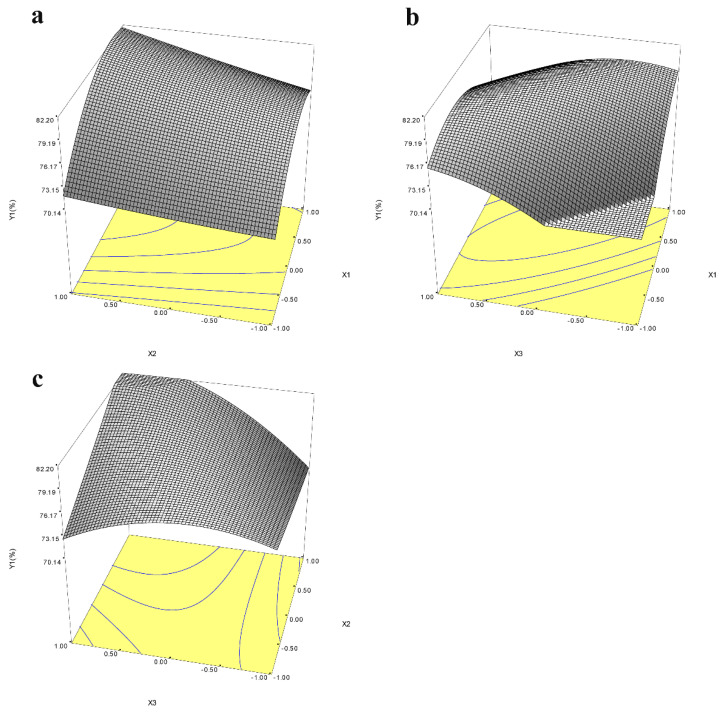
Response surface maps of the interaction of enzyme activity (*X*_1_) and pH (*X*_2_) (**a**), enzyme activity (*X*_1_) and hydrolysis time (*X*_3_) (**b**), and pH (*X*_2_) and hydrolysis time (*X*_3_) (**c**) on icariin yield (*Y*_1_). The coordinate *X* represents the code value, not the actual value.

For optimization of the yield of icariin and epimedin C remaining, BBD was performed by three factors, including time, pH, and the amount of α-L-rhamnosidase, based on the above results. The runs and the value of icariin yield were shown in Table 1. Analysis of variance (ANOVA) for the response surface quadratic model for time, pH, and the amount of α-L-rhamnosidase were presented in Table 3 and Table S1 to estimate the statistical significance of the coefficients of the model. The quadratic model between icariin yield and the independent variables could be established by the following equation: *Y*_1_ (icariin yield) = 78.500 + 3.866*X*_1_ + 1.941*X*_2_ + 2.432*X*_3_ − 3.733*X*_1_ *× X*_1_ + 0.171*X*_2_ *× X*_2_ − 2.784*X*_3_ *× X*_3_ + 1.005*X*_1_ *× X*_2_ − 5.137*X*_1_ *× X*_3_ + 3.628*X*_2_ *×* *X*_3_. In the ANOVA table, *F*-value and *p*-value are applied to determine significance of the regression model for each response selected. If the model possesses a high *F*-value and the *p*-value is less than 0.05, it is considered to be significant [32]. The *F*-value for the quadratic model of icariin yield showed a value of 4.714 and the *p*-value was 0.027, which indicated that the build the quadratic model for icariin yield was significant (Table 3). The *p*-value of “Lack of Fit” was 0.074 (>0.05) indicating that the built regression equation is appropriate. Figure S4 showed the response surface map of enzyme dosage, pH, and time. The response surface of enzyme activity and pH, and the response surface of enzyme activity and time had a steep slope and dense contour line, indicating that the interaction between enzyme amount and pH or time had significant influence on the increase in icariin yield (Figure 6a,b). The quadratic model for epimedin C remaining was also significant because the *p*-value of the model was 0.0002 (<0.05) and *p*-value of “Lack of Fit” is 0.055 (>0.05) (Table S1). The response surface map of enzyme dosage, pH, and time for epimedin C remaining was presented in the Figure S4. When two response values were optimized together, the maximum values of icariin yield and epimedin C remaining were 83.89% and 3.89% respectively, at pH 7.5 with 6.528 U of enzyme in 2 mL reaction volume for 9 h. Three batches of fermentation experiments were performed under the controlled conditions with a temperature of 45 °C, pH 7.5, 6.56 U of enzyme, and reaction time of 9 h, and the mean of icariin yield was 83.28 ± 0.75%, which was not significantly different from the predicted value. Thus, the model was perceived to be reliable for predicting the yield of icariin by the hydrolysis of *E*. *wanshanense* Ying water extract using α-L-rhamnosidase from *P. laurentii* ZJU-L07. After response surface optimization, the yield of icariin did not increase significantly, but the amount of enzyme required dropped from 19.13 U to 6.56 U, which reduced the cost of production.

## 4. Discussion

As an important biotechnology enzyme, α-L-rhamnosidase has been paid attention because it could hydrolyze various natural compounds. With the pharmacological effects of icariin being paid more and more attention, the preparation of icariin by α-L-rhamnosidase has also been gradually researched. Different sources of α-L-rhamnosidase have different abilities to hydrolyze different glycosidic bonds [15]. It was reported that α-L-rhamnosidase from *B. thetaiotaomicron* [13], *A. nidulans* [14], and *T. petrophila* [23] could convert epimedin C into icariin, but it was rarely reported that α-L-rhamnosidase from yeast could hydrolyze epimedin C. In this study, α-L-rhamnosidase from *P. laurentii* ZJU-L07 could hydrolyze the rhamnoside-α-1,2-rhamnoside glycosidic bond and transform epimedin C into icariin. Moreover, α-L-rhamnosidase from *P. laurentii* ZJU-L07 could also hydrolyze the glucoside-α-1,2-rhamnoside glycosidic bond and transform neohesperidin and naringin into hesperetin-7-O-glucoside and prunin, respectively. Therefore, α-L-rhamnosidase from *P. laurentii* ZJU-L07 could also be used for debittering citrus juices [33] and the synthesis of hesperetin 7-O-glucoside [20]. In previous reports, some yeast-derived α-L-rhamnosidases have also been studied, such *Saccharomyces cerevisiae*, *Hanshula anomala*, *Debaryomyces ploymorphus* [34], *C. albidus* [35], and *P. angusta* X349 [26]. *S. cerevisiae*, *H. anomala*, and *D. ploymorphus* show low levels of α-L-rhamnosidase activities, which are not good sources of α-L-rhamnosidase production. α-L-rhamnosidase from *C. albidus* [35] could transform neohesperidin and naringin, and α-L-rhamnosidase from *P. angusta* X349 [26] could hydrolyze naringin, hesperidin, rutin, and 3-quercitrin, which can be further studied to produce α-L-rhamnosidase for the conversion of some natural products.

The efficiency of enzyme conversion of natural products is not only related to the specificity of the enzyme, but also related to the activity of the enzyme. Enzymatic properties show the effects of temperature, pH, and metal ions on enzyme activity. In this study, the optimum temperature of α-L-rhamnosidase was 55 °C (Figure 3a), which was identical to that of α-L-rhamnosidase from *A*. *nidulan* [14] and *B*. *thetaiotaomicron* [13], lower than that of *C*. *albidus* (60 °C) [35] and *T. petrophila* DSM 13995 (90 °C) [23], and higher than of *P. angusta* X349 (40 °C) [26], *A. flavus* MTCC-9606 (50 °C) [36], *A. aculeatus* JMUdb058 (50 °C) [33], and *B. amyloliquefaciens* 11568 (45 °C) [37]. However, the optimum temperature for transforming epimedin C was 45 °C and the catalytic efficiency was not high at 55 °C, which was lower than at 40, 45, and 50 °C (Figure 5c). This may be related to the thermal stability of α-L-rhamnosidase (Figure 3b). α-L-Rhamnosidase had poor thermal stability at 55 °C as shown in Figure 3b, and the enzyme activity was gradually lost upon higher temperature. Therefore, after 3 h of incubation at 55 °C, the amount of icariin hardly increased (Figure 5c). Therefore, the optimal temperature of the enzyme is not necessarily a suitable temperature for long-term enzymatic hydrolysis. This is similar to α-L-rhamnosidase from *A. alternata* SK37.001. The optimal temperature of α-L-rhamnosidase from *A. alternata* SK37.001 was 60 °C, and it was relatively stable at a temperature between 30 and 50 °C [16]. However, when the enzyme was kept at 60 °C for a long time, its activity decreased rapidly [16]. The optimum pH of α-L-rhamnosidase was 7 (Figure 3c). Moreover, when compared with pH 6 and 6.5, 7 was also the optimal pH for hydrolyzing epimedin C (Figure 3f). The optimal pH of the reported α-L-rhamnosidases ranged from 2.0 to 11.0 and varied between microorganisms [15,36]. The optimum pH of most fungal-derived α-L-rhamnosidases were acidic, while the optimal pH of most bacterial-derived α-L-rhamnosidases were alkaline [15]. Of course, there were some exceptions [19,38], and the optimal pH of α-L-rhamnosidase from *F. moniliforme* MTCC-2088 was 10.5 [38], and some were neutral, such as the α-L-rhamnosidase studied in this work, α-L-rhamnosidases from *Aspergillus awamori* MTCC-2879 [39] and *Penicillium griseoroseum* MTCC-9224 [40]. *K_m_* means the affinity of enzyme and substrate, and the larger the value of *K_m_* means the smaller the affinity between enzyme and substrate. Metal ions also affected enzyme activity. Among them, Fe^2+^ and Cu^2+^ had a strong inhibitory effect on enzymes, which might destroy the active group of the enzyme [35]. This was similar to the properties of α-L-rhamnosidase from *A. alternata* SK37.001 [16], *P. citrinum* MTCC-8897 [40], and *B. thetaiotaomicron* [13], and Cu^2+^ had extremely high levels of rhamnosidase inhibition. The *K_m_* of the α-L-rhamnosidase studied with pNPR as the substrate was lower than that of α-L-rhamnosidase from some other microorganisms, which indicated that the affinity of the enzyme for pNPR was superior to that of other-sourced enzymes [13,16,31]. *K_m_* of α-L-rhamnosidase with epimedin C as the substrate was higher than that of α-L-rhamnosidase from *A. nidulans* and *T. petrophila* [14,23], which indicated that the affinity of the enzyme for epimedin C was slightly worse than that of other-sourced enzymes.

In all the experiments of enzymatic hydrolysis of epimedin C, epimedin C in the reaction system was not completely converted to icariin. The crude enzyme not only contained α-L-rhamnosidase, but also other enzymes. α-L-Rhamnosidase and β-D-glucosidase usually coexist in microorganisms. Naringinase is an enzyme complex that possesses the activities of both α-L-rhamnosidase and β-D-glucosidase, which cleave terminal α-L-rhamnose and β-D-glucose from flavonoids and other products [33]. β-D-Glucosidase was proved to exist in the crude enzyme using pNPG as the substrate. Epimedin C was transformed into sagittatoside C by β-D-glucosidase and transformed into icariside II by α-L-rhamnosidase and β-D-glucosidase [23]. This was also why the yield of icariin first increased and then decreased when the enzyme activity was 8.79 U or 19.13 U. When the activity of α-L-rhamnosidase in the crude enzyme increased, the enzyme activity of β-D-glucosidase would also increase, whose hydrolysis effect would be more obvious with the increase in time. Therefore, an appropriate amount of enzyme was the key to increasing the content of icariin. Adding excessive amounts of enzyme not only raised the production cost, but also led to excessive hydrolysis of epimedin C, while a small amount of enzyme would cause epimedin C to not be completely converted into icariin. After response surface optimization, 6.528 U of enzyme in 2 mL reaction volume was a suitable amount of enzyme. This would provide a reference for the expansion of icariin production.

## 5. Conclusions

In summary, icariin as a chemical marker for the quality control of Herba Epimedii in Chinese Pharmacopeia [41], has extensive pharmacological effects. However, the resources of icariin in nature are rare and α-L-rhamnosidase transforming epimedin C into icariin is rarely reported. In this work, a strain with bioconversion epimedin C to icariin was successfully screened from the soil and named as *P*. *laurentii* ZJU-L07. α-L-Rhamnosidase from *P. laurentii* ZJU-L07 has a highly efficient enzymatic hydrolysis effect on epimedin C. When the concentration of epimedin C in the reaction systems was 500 mg/L, 87% and 85% of epimedin C in standard epimedin C and EWE-I were converted into icariin, respectively. After optimizing the reaction conditions, more than 83% of epimedin C in EWE-II was converted into icariin. The efficient and highly specific α-L-rhamnosidase provides a valuable protocol for industrialized production of icariin in the future. Nevertheless, there are some difficulties in large-scale preparation because this enzyme is an intracellular enzyme. Therefore, in future studies, an α-L-rhamnosidase gene from *P. laurentii* ZJU-L07 could be cloned and expressed in *P*. *pastoris* [42]. Alternatively, the enzyme could be immobilized to improve enzyme utilization.

## Figures and Tables

**Table 1:** Box–Behnken design matrix for optimization of the yield of icariin production.

Run	*X*_1_ Enzyme Dosage (U)	*X*_2_ pH	*X*_3_ Hydrolysis Time (h)	*Y*_1_ Yield of Icariin (%)	*Y*_2_ Epimedin C Remaining (%)
1	−1 (3.84)	−1 (6.5)	0 (6)	72.29	16.01
2	0 (6.64)	1 (7.5)	−1 (3)	74.34	16.82
3	−1	0 (7.0)	1 (9)	76.11	8.13
4	0	0	0	79.70	3.99
5	0	0	0	80.24	3.99
6	1 (9.53)	0	−1	78.13	10.74
7	0	−1	1	70.18	4.86
8	1	1	0	79.60	5.48
9	−1	1	0	73.21	19.40
10	0	0	0	79.56	6.32
11	0	0	0	77.49	6.68
12	−1	0	−1	56.77	38.86
13	1	−1	0	74.65	6.19
14	0	1	1	82.26	5.39
15	0	0	0	75.51	3.99
16	0	−1	−1	76.77	16.47
17	1	0	1	76.92	3.99

**Table 2:** Substrate specificity of α-L-rhamnosidase.

Substrate	Hydrolyzable Bonds	Product Formed	Yield (%)
Epimedin C	α-1,2	Icariin	60.0
Neohesperidin	α-1,2	Hesperetin-7-O-glucoside	61.3
Naringin	α-1,2	Prunin	59.5
Hesperidin	α-1,6	Hesperetin-7-O-glucoside	4.5
Rutin	α-1,6	Quercetin-3-β-D-glucoside	6.6

**Table 3:** ANOVA of the quadratic model for the yield of icariin.

Source	Sum of Squares	Degree of Freedom	Mean Square	*F*-Value	*p*-Value Probability > *F*	Remarks
Model	455.642	9	50.627	4.714	0.027	significant
*X*_1_	119.597	1	119.597	11.136	0.013	
*X*_2_	30.146	1	30.146	2.807	0.138	
*X*_3_	47.317	1	47.317	4.406	0.074	
*X*_1_^2^	58.678	1	58.678	5.463	0.052	
*X*_2_^2^	0.124	1	0.124	0.012	0.918	
*X*_3_^2^	32.637	1	32.637	3.039	0.125	
*X*_1_ × *X*_2_	4.043	1	4.0427	0.376	0.559	
*X*_1_ × *X*_3_	105.545	1	105.545	9. 263	0.017	
*X*_2_ × *X*_3_	52.654	1	52.654	4.903	0.062	
Residual	75.180	7	10.740			
Lack of Fit	59.644	3	19.881	5.119	0.074	not significant
Pure Error	15.536	4	3.884			
Cor Total	530.822	16				

## Data Availability

The data presented in this study are available in article and Supplementary Material.

## Supplementary Materials

The following supporting information can be downloaded at: https://www.mdpi.com/article/10.3390/jof8060644/s1, Figure S1: Strain screening results after mutagenesis for producing α-L-rhamnosidase transforming epimedin C. (a) Strains on LB medium including pNPR, (b) Icariin Yield, (c) The morphology of *P. laurentii* ZJU-L07 on YPD medium. Figure S2: Chromatographs of the reaction mixtures before and after enzymatic hydrolysis of hesperidin (a), rutin (b), naringin (c), neohesperidin (d) and epimedin C (e). Figure S3: Enzymatic hydrolysis of EWE-II. (a) The amount of α-L-rhamnosidase on Epimedin C remained at 50 °C under pH 4.0 with 3.95 U of enzyme. (b) The amount of α-L-rhamnosidase on Epimedin C remained at 50 °C under pH 4.0 with 500 mg·L^−1^ epimedin C. (c) Temperature affection with 3.95 U of enzyme in the reaction system containing 500 mg·L^−1^ epimedin C at pH 7.0. (d) pH affection with 3.95 U of enzyme in the reaction system containing 500 mg·L^−1^ epimedin C at 50 °C. Figure S4: Response surface maps of the interaction of enzyme activity (*X*_1_) and pH (*X*_2_) (a), enzyme activity (*X*_1_) and hydrolysis time (*X*_3_) (b), and pH (*X*_2_) and hydrolysis time (*X*_3_) (c) on Epimedin C remained (*Y*_2_). The coordinate *X* represents the code value, not the actual value. Table S1: ANOVA of the quadratic model for Epimedin C remained.
