# Supplementary material for: Purification and Characterization of a Novel α-L-Rhamnosidase from Papiliotrema laurentii ZJU-L07 and Its Application in Production of Icariin from Epimedin C
Source: J Fungi (Basel). 2022 Jun 20;8(6):644. doi: 10.3390/jof8060644 (PMC9225045; doi:10.3390/jof8060644)
Supplement: Supplementary file 1 [file jof-08-00644-s001.zip › jof-1758468-supplementary.pdf]

Supplementary File 1

1. Materials and Methods

The calculation method of icariin yield and epimedin C is as follows.

$$\text{Icariin Yield (\%)} = \frac{\Delta m \times Mr_1}{m_1 \times Mr_2} \times 100$$

$$\text{Epimedin C remained (\%)} = \frac{m_2}{m_1} \times 100$$

∆m: Mass difference of icariin before and after reaction.

m1: The quality of epimedin C before the reaction.

m2: The quality of epimedin C after the reaction.

Mr1: Relative molecular mass of epimedin C.

Mr2: Relative molecular mass of icariin.

2. Results

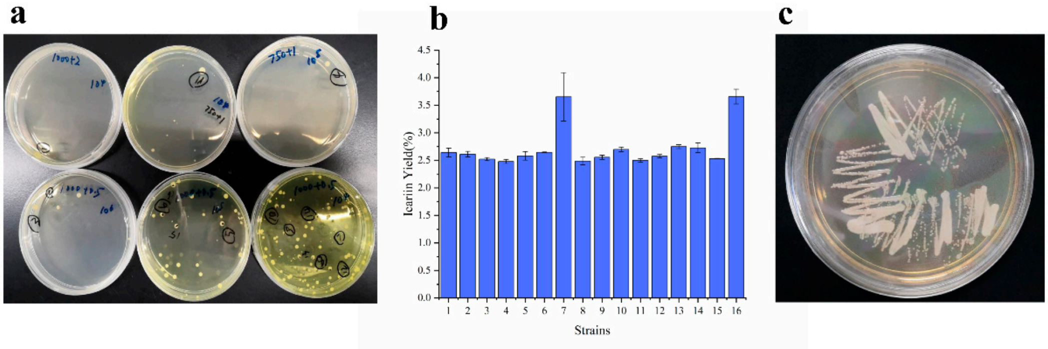

Figure S1 Strain screening results after mutagenesis for producing α-L-rhamnosidase transforming epimedin C. (a) Strains on LB medium including pNPR, (b) Icariin Yield, (c) The morphology of *P. laurentii* ZJU-L07 on YPD medium

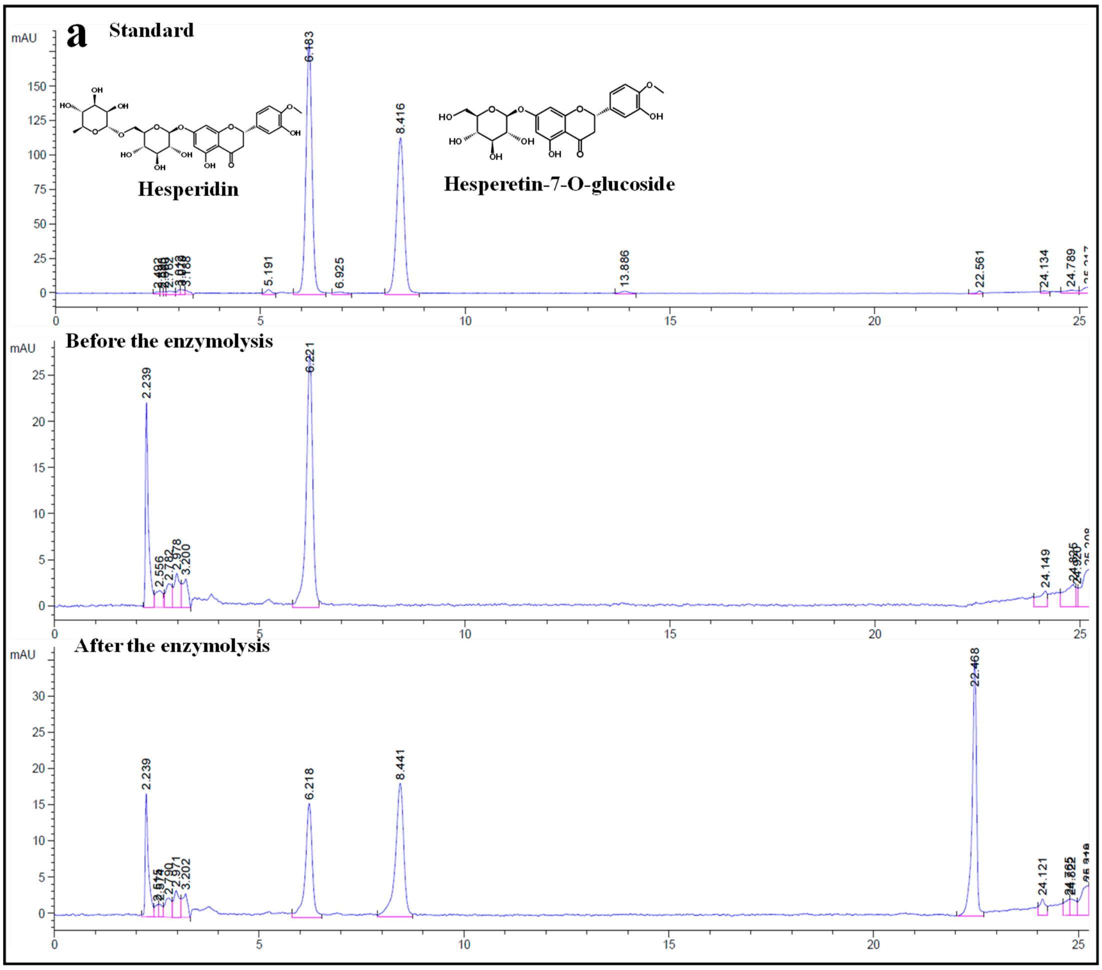
a
Standard
Hesperidin
Hesperetin-7-O-glucoside
Before the enzymolysis
After the enzymolysis
mAU
6.183
8.416
2.239
6.221
6.218
8.441
22.468

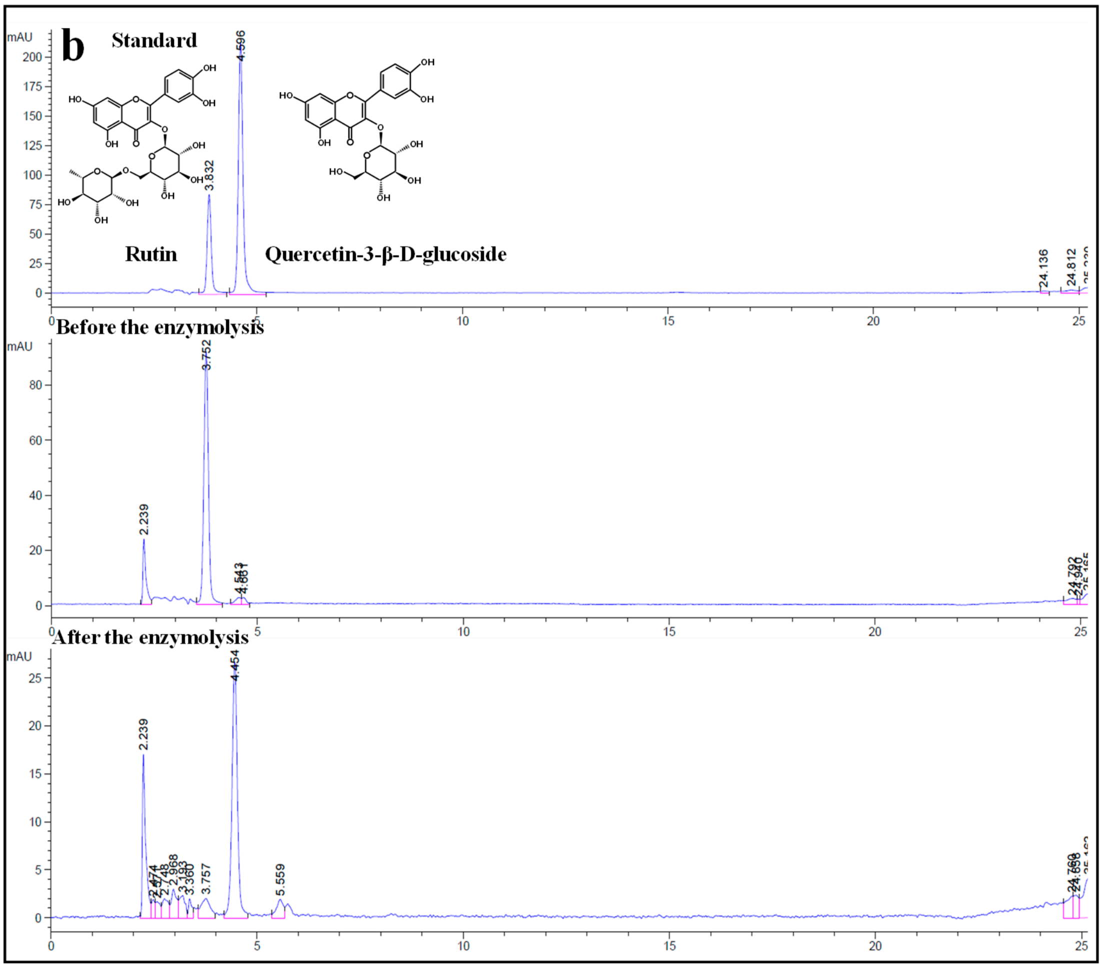
b
Standard
Rutin
Quercetin-3-β-D-glucoside
Before the enzymolysis
After the enzymolysis
mAU
3.832
4.596
2.239
3.752
4.454
5.559
3.757

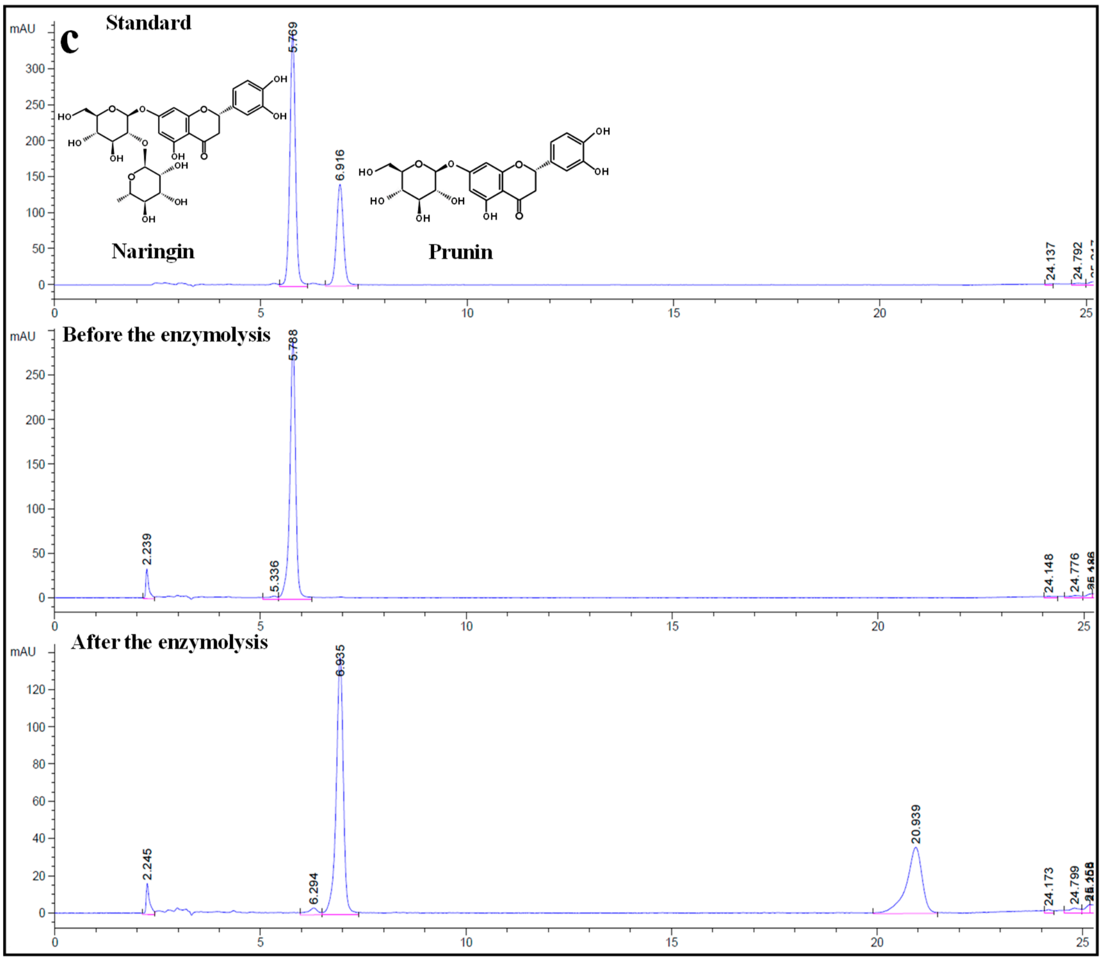
c
Standard
Naringin
Prunin
Before the enzymolysis
After the enzymolysis
mAU
5.769
6.916
5.788
2.239
5.336
6.935
2.245
6.294
20.939
24.137
24.792
24.148
24.776
24.173
24.799

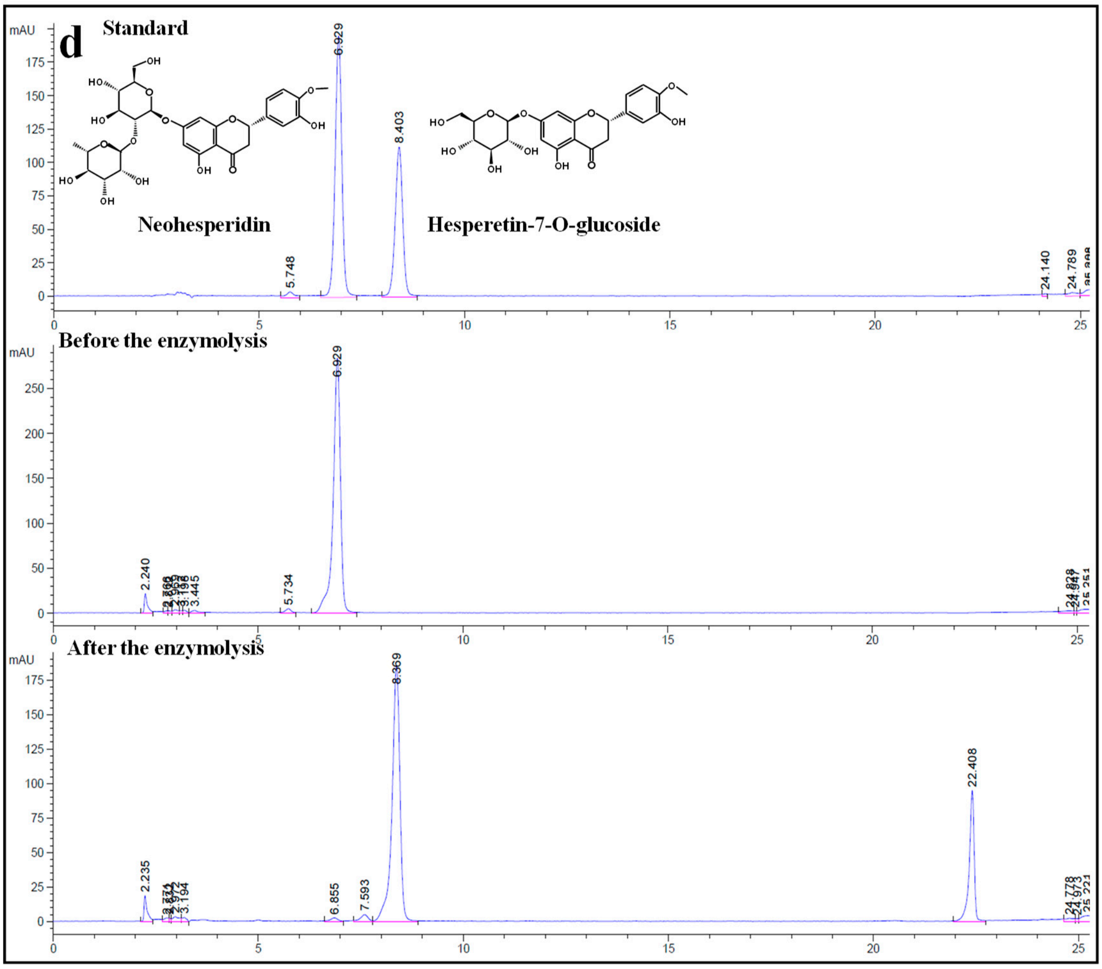
d
Standard
Neohesperidin
Hesperetin-7-O-glucoside
Before the enzymolysis
After the enzymolysis
mAU
6.929
8.403
5.748
24.140
24.789
2.240
3.445
5.734
2.235
3.194
6.855
7.593
8.369
22.408

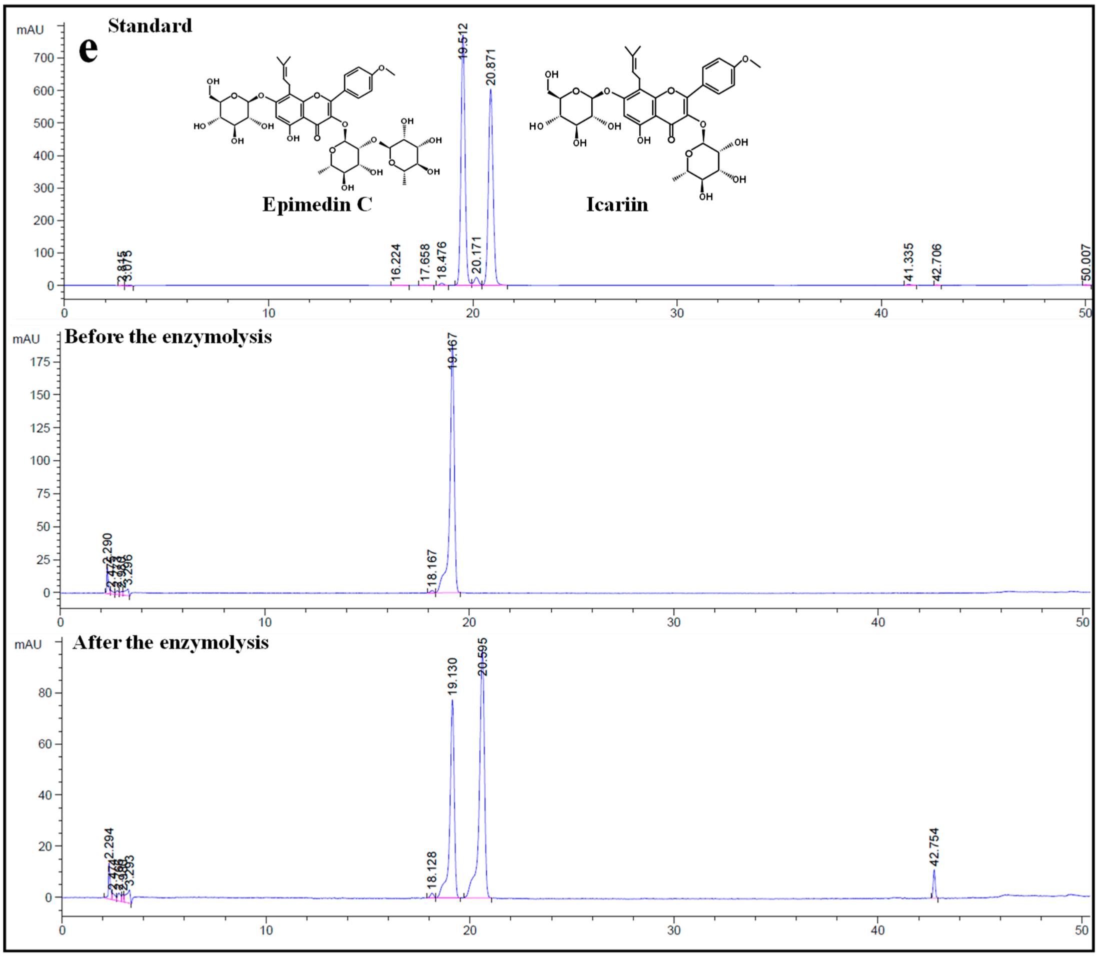

Figure S2 Chromatographs of the reaction mixtures before and after enzymatic hydrolysis of hesperidin(a), rutin(b), naringin(c), neohesperidin(d) and epimedin C(e).

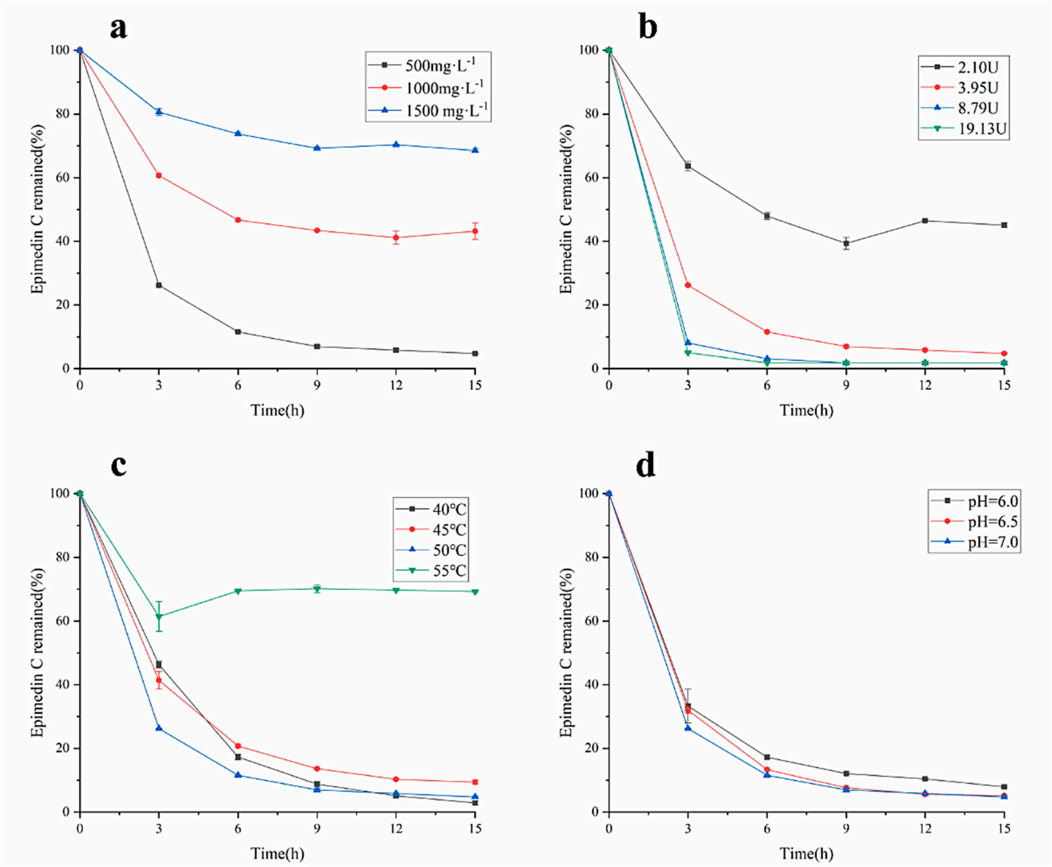

Figure S3 Enzymatic hydrolysis of EWE-II. (a) The amount of α-L-rhamnosidase on Epimedin C remained at 50 °C under pH 4.0 with 3.95 U of enzyme. (b) The amount of α-L-rhamnosidase on Epimedin C remained at 50 °C under pH 4.0 with 500 mg·L^{-1} epimedin C. (c) Temperature affection with 3.95U of enzyme in the reaction system containing 500 mg·L^{-1} epimedin C at pH 7.0. (d) pH affection with 3.95U of enzyme in the reaction system containing 500 mg·L^{-1} epimedin C at 50 °C.

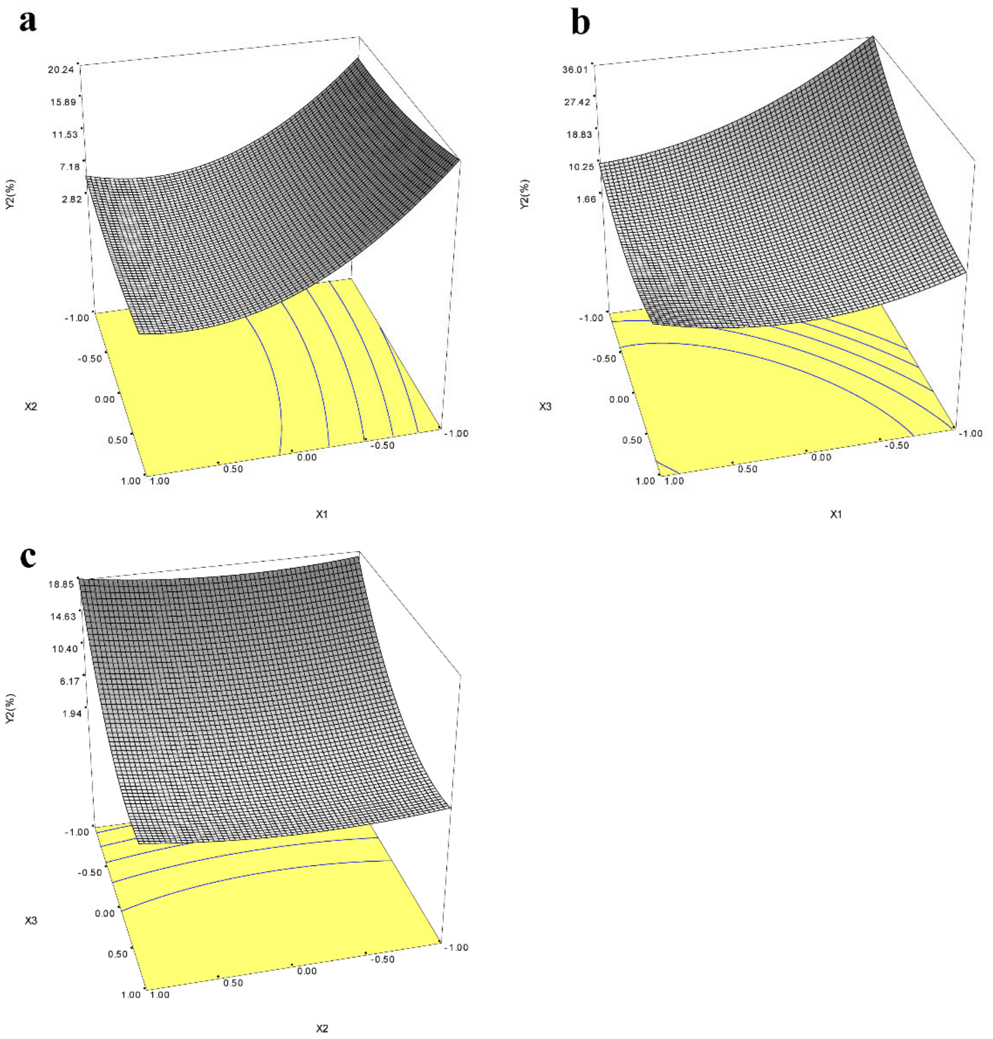

Figure S4 Response surface map of enzyme dosage (*X_1*), pH (*X_2*) and time (*X_3*) for Epimedin C remained (*Y_2*). Response surface maps of the interaction of enzyme activity (*X_1*) and pH (*X_2*) (a), enzyme activity (*X_1*) and hydrolysis time (*X_3*) (b), and pH (*X_2*) and hydrolysis time (*X_3*) (c) on Epimedin C remained (*Y_2*). The coordinate *X* represents the code value, not the actual value.

Table S1 ANOVA of the quadratic model for Epimedin C remained

Source	Sum of Squares	Degree of freedom	Mean Square	F- Value	p-value Probability > F	Remarks
Model	1256.579	9	139.620	22.529	0.0002	significant
X_1	392.305	1	392.3052	63.302	< 0.0001	
X_2	1.586	1	1.586	0.256	0.628	
X_3	458.441	1	458.441	73.973	< 0.0001	
X_1^2	135.017	1	135.017	21.786	0.0023	
X_2^2	5.220	1	5.220	0.842	0.389	
X_3^2	96.122	1	96.122	15.510	0.006	
$X_1 \times X_2$	4.195	1	4.195	0.677	0.438	
$X_1 \times X_3$	144.090	1	144.090	23.250	0.002	
$X_2 \times X_3$	0.009	1	0.009	0.0014	0.971	
Residual	43.382	7	6.197			
Lack of Fit	35.702	3	11.901	6.199	0.055	not significant
Pure Error	7.679	4	1.920			
Cor Total	1299.961	16				
